# Intranasal Flunisolide Suppresses Pathological Alterations Caused by Silica Particles in the Lungs of Mice

**DOI:** 10.3389/fendo.2020.00388

**Published:** 2020-06-17

**Authors:** Tatiana Paula Teixeira Ferreira, Januário Gomes Mourão e Lima, Francisco Alves Farias-Filho, Yago Amigo Pinho Jannini de Sá, Ana Carolina Santos de Arantes, Fernanda Verdini Guimarães, Vinicius de Frias Carvalho, Cory Hogaboam, John Wallace, Marco Aurélio Martins, Patrícia Machado Rodrigues e Silva

**Affiliations:** ^1^Laboratory of Inflammation, Oswaldo Cruz Institute, Oswaldo Cruz Foundation, Rio de Janeiro, Brazil; ^2^Department of Medicine, Cedars-Sinai Medical Center, Women's Guild Lung Institute, Los Angeles, CA, United States; ^3^Departments of Physiology and Pharmacology, and Medicine, Cumming School of Medicine, University of Calgary, Calgary, AB, Canada

**Keywords:** lung, fibrosis, silica particles, therapy, flunisolide

## Abstract

Silicosis is an occupational disease triggered by the inhalation of fine particles of crystalline silica and characterized by inflammation and scarring in the form of nodular lesions in the lungs. In spite of the therapeutic arsenal currently available, there is no specific treatment for the disease. Flunisolide is a potent corticosteroid shown to be effective for controlling chronic lung inflammatory diseases. In this study, the effect of flunisolide on silica-induced lung pathological changes in mice was investigated. Swiss-Webster mice were injected intranasally with silica particles and further treated with flunisolide from day 21 to 27 post-silica challenge. Lung function was assessed by whole body invasive plethysmography. Granuloma formation was evaluated morphometrically, collagen deposition by Picrus sirius staining and quantitated by Sircol. Chemokines and cytokines were evaluated using enzyme-linked immunosorbent assay. The sensitivity of lung fibroblasts was also examined in *in vitro* assays. Silica challenge led to increased leukocyte numbers (mononuclear cells and neutrophils) as well as production of the chemokine KC/CXCL-1 and the cytokines TNF-α and TGF-β in the bronchoalveolar lavage. These alterations paralleled to progressive granuloma formation, collagen deposition and impairment of lung function. Therapeutic administration of intranasal flunisolide inhibited granuloma and fibrotic responses, noted 28 days after silica challenge. The upregulation of MIP-1α/CCL-3 and MIP-2/CXCL-2 and the cytokines TNF-α and TGF-β, as well as deposition of collagen and airway hyper-reactivity to methacholine were shown to be clearly sensitive to flunisolide, as compared to silica-challenge untreated mice. Additionally, flunisolide effectively suppressed the responses of proliferation and MCP-1/CCL-2 production from IL-13 stimulated lung fibroblasts from silica- or saline-challenged mice. In conclusion, we report that intranasal treatment with the corticosteroid flunisolide showed protective properties on pathological features triggered by silica particles in mice, suggesting that the compound may constitute a promising strategy for the treatment of silicosis.

## Introduction

Silicosis is a work-related and occupational disease caused by long-term exposure to inhaled dust containing crystalline silica particles which can progress to severe lung inflammation and fibrosis (1). There are three forms of silicosis (acute, accelerated, and chronic) dependent on the amount and time of silica particle exposure (2), which can be complicated by increased risk of infections (3) and/or chronic obstructive pulmonary disease (4). It became a greater public health problem worldwide after the Industrial Revolution, which increased dust levels and the number of workers exposed around the world (5). Among activities with elevated risk are those involving sandblasting, brickworks, civil construction, mining and many others (6).

Silicosis persists mainly in developing countries due to a lack of application of protective measures against environmental dust (5). Existing evidence shows that about 6 million workers are exposed to silica in Brazil, 11.5 million in India and 23 million are at risk of getting the disease in China, and more than 24 thousand deaths are reported annually (6–8). The incidence of silicosis is also on the rise in workers of the stonecutting industry in Australia (9) and sandblast fashion denim in Turkey (10). Since no effective treatment exists for silicosis currently, new therapies are badly needed in this field (1).

It is noteworthy that daily oral prednisolone therapy suppressed alveolitis in some patients with chronic silicosis, improving lung function and gas exchange, under conditions where responders and non-responders did not differ with regard to simple or complicated silicosis (11). However, systemic use of corticosteroids is often associated with significant adverse effects such as pituitary-adrenal suppression, cataract formation, hypertension and osteoporosis, among others (12). Flunisolide is a well-known intranasal corticosteroid molecule reported to be effective in inhibiting adverse remodeling in the peripheral airways of mild and moderate asthmatic patients by reducing expression the α-SMA (13). It also inhibited idiopathic pulmonary hemosiderosis, a rare disease characterized by bleeding into the pulmonary alveoli and progressive lung fibrosis (14).

Thus, considering the lack of an appropriate therapy for silicotic patients, in the present study we evaluated the effectiveness of intranasal administration of flunisolide on the chronic pulmonary inflammation caused by instillation of silica particles into mice.

## Materials and Methods

### Animals

Male Swiss Webster mice (18–20 g) were obtained from the Oswaldo Cruz Foundation (Rio de Janeiro, Brazil) breeding unit and kept in the care facility of Oswaldo Cruz Institute. Animals were kept in ventilated cages (in groups of five) at 22–25°C and relative humidity (40–70%), on a 12 h light/dark cycle with food and water *ad libitum*. All the animal experiments were conducted in accordance with the guidelines of the Committee on Use of Laboratory Animals of the Oswaldo Cruz Foundation (license LW057/14).

### Silicosis Induction and Treatment

**Figure 1:**
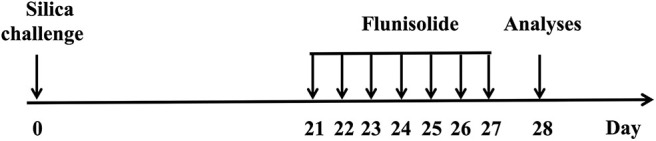
Schematic study protocol for induction of silicosis and treatment in mice. Silica particles (10 mg/mice) were instilled intranasally, and animals were treated with intranasal flunisolide (0.3–10 μg/mouse) once a day from days 21 to 27 post-silica challenge. The analyses were performed on day 28.

Animals were anesthetized with isofluorane (5%) (Cristália, São Paulo) and then instilled, intranasally, with crystalline silica (10 mg/50 μL/mouse) (particle size 0.5–10 μm; Sigma Chemical Co, St. Louis, MO) diluted in sterile 0.9% NaCl (15). Sham-challenged mice were instilled with similar volume of 0.9% NaCl. Analyses were performed at 7, 14, and 28 days post-silica challenge. Flunisolide (0.3–10 μg/mouse) was dissolved in 0.9% NaCl and intranasally administered, daily, from days 21–27 and analyses were performed on day 28 (Figure 1). Silica-challenged mice received the same volume (20 μL) of the treatment vehicle.

### Bronchoalveolar Lavage (BAL)

Animals were killed with sodium pentobarbital (500 mg/kg, i.p.), and the bronchoalveolar lavage (BAL) was performed as previously described (16). Total leukocytes were counted in a Neubauer chamber and differential cell counts performed in cytospin preparations stained with May-Grunwald-Giemsa dye. Analyses were made under light microscopy (BX50, Olympus).

### Lung Histology

After bronchoalveolar lavage and lung perfusion, the left lung was removed and fixed in Milloning buffer solution (pH 7.4) with 4% paraformaldehyde, and sections of 4 μm were stained with hematoxylin & eosin (H&E) or Picrus sirius. Lung morphometric analysis was performed by an integrating eyepiece with a coherent system consisting of a grid with 100 points and 50 lines (known length) (17) coupled to a light microscope (Olympus BX50), connected to a video camera (Optronics Engineering, DEI-750). The camera output was processed and examined by image analyzer software Image-Pro Plus Version 4. Silica crystals were quantitatively analyzed, in 15 independent fields, with a light microscope (Olympus BX50) equipped with polarizing attachment for detecting birefringent particles and Image-Pro Plus Version 4. The results were expressed as number of pixels per μm^2^ of tissue. To avoid experimental bias, the slides were evaluated in blind fashion.

### Immunohistochemistry

Left lung tissue samples were evaluated for immunohistochemical localization of F4/80 and α-SMA (α-smooth muscle actin). The antibodies were obtained from the following sources: anti-mouse F4/80 (MCA497G) from AbD Serotec (Kidlington, UK) and anti-α-SMA (A2547) from Sigma-Aldrich (St. Louis, USA). Secondary antibodies conjugated with horseradish peroxidase (HRP) were all obtained from R&D Systems (Minneapolis, USA). To determine the specificity of staining, no primary antibody was used followed by incubation of section with secondary antibodies and detection reagents (18).

### Quantification of Lung Collagen

Right lung was homogenized in Tris-HCl 0.05M, NaCl 1M containing protease inhibitor cocktail (Hoffmann-La Roche Ltd, Switzerland) (pH = 7.4). Total soluble collagen was extracted overnight at 4°C and quantification performed using the Sircol™ kit (Biocolor Ltd, Newton Abbey, UK). Results were expressed as mg of collagen per right lung.

### ELISA Analysis

The concentration of murine KC/CXCL-1, MIP-2/CXCL-2, MIP-1α/CCL-3, MCP-1/CCL-2, TNF-α and TGF-β, was measured in the supernatant of BAL and/or in homogenates from right lung samples by means of ELISA as previously described (15). For lung tissue, samples were homogenized in PBS containing 0.05% Triton X-100 and protease inhibitor cocktail (Hoffmann-La Roche, Basel, Switzerland). Reagents from commercial DuoSet kits (R&D Systems, Minneapolis, MN, USA) were used in accordance with the instructions of the manufacturer.

### Invasive Assessment of Respiratory Mechanics

Mice were anesthetized with nembutal (60 mg/kg) and the neuromuscular activity was blocked with bromide pancuronium (1 mg/kg). Tracheostomized animals were mechanically ventilated and the lung function assessed. Airway resistance (cmH_2_O.s/mL) and lung elastance (mL/cmH_2_O) were assessed using a FinePointe R/C Buxco Platform (DSI^TM^, Minneapolis) (15). Animals were allowed to stabilize for 5 min and increasing concentrations of methacholine (3–81 mg/mL) were aerosolized for 5 min each. Baseline pulmonary parameters were assessed with aerosolized PBS.

### Lung Fibroblast Isolation and Activation

The whole lung was removed from mice, 7 d after silica-challenge, perfused under aseptic conditions and gently dispersed on steel mesh followed by enzymatic digestion with collagenase 1A (1 mg/mL in 10 mL) (Sigma-Aldrich) for 1 h at 37 C. Lung fibroblasts from saline-challenged mice were used as control group. Dispersed cells were submitted to a continuous Percoll gradient (GE Healthcare) to eliminate silica particles and then placed in DMEM medium plus 10% of FBS, 1% penicillin-streptomycin at 37 C, and 5% CO2. Cells were stimulated with rmIL-13 (40 ng/mL), for 24 h. After centrifugation, 0.25 × 10^6^ cells were added to 6-well plates, incubated with flunisolide (0.1–100 μM) at 37°C and a 5% CO_2_, for 24 h. Proliferation was assessed via [^3^H] thymidine incorporation (0.5 mCi/well). In another set of experiments, after centrifugation, the supernatant was recovered and quantified for chemokine MCP-1/CCL2 by ELISA.

### Statistical Analysis

Results were expressed as mean ± standard error of mean (SEM) and statistical analysis was done with one-way ANOVA followed by the multiple comparison test of Newman-Keuls-Student. Values of *p* < 0.05 were considered statistically significant for both tests.

## Results

### Time Course of the Airways Inflammation Caused by Exposure to Silica in Mice

**Figure 2:**
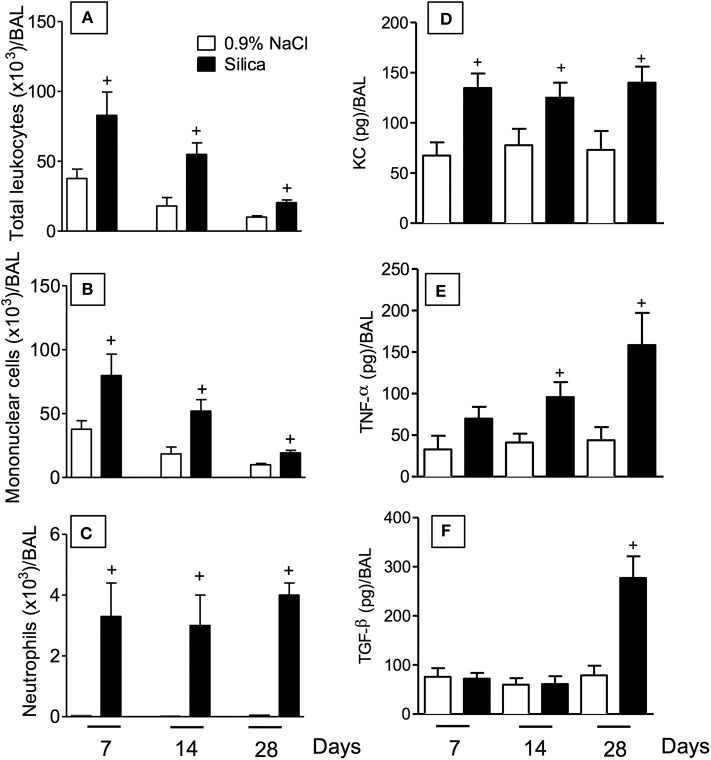
Inflammatory changes in BAL effluent following silica particle instillation. BAL effluent samples were obtained from sham-challenged mice (open column) or silica-challenged mice (closed column) for quantification of total leukocytes **(A)**, mononuclear cells **(B)**, neutrophils **(C)**, KC/CXCL1 **(D)**, TNF-α **(E)**, and TGF-β **(F)** on days 7, 14, and 28. Values represent mean ± SEM from 5 animals per group. Statistical analysis was done with one-way ANOVA followed by Newman-Keuls-Student test. ^+^*P* < 0.05 as compared to saline-challenged animals.

Intranasal instillation of crystaline silica particle suspension in mice resulted in an increased number of leukocytes in the bronchoalveolar space at days 7, 14, and 28 post-challenge as compared to sham-challenged mice (Figure 2A). Although the maximal response was at day 7 post-silica, the absolute number of leukocytes remained significantly increased over the saline-challenged mice at days 14 and 28 (Figure 2A). Standard staining of cytospin preparations revealed a predominant mononuclear cell infiltration into the airways after silica challenge (Figure 2B), while a slight but significant elevation in polymorphonuclear neutrophil counts was noted from 7 to 28 days (Figure 2C). The profile of chemokines and cytokines was also investigated. As shown in Figure 2D, KC/CXCL-1 levels in the BAL effluent appeared significantly increased at all timepoints analyzed, in a clear association with the polymorphonuclear neutrophl influx (Figure 2C). In contrast, levels of the cytokines TNF-α and TGF-β appeared elevated at later timepoints, day 14 and 28 concerning the former (Figure 2E) and only at day 28 (Figure 2F) concerning the latter.

### Time Course of Lung Pathological Changes Caused by Exposure to Silica in Mice

**Figure 3:**
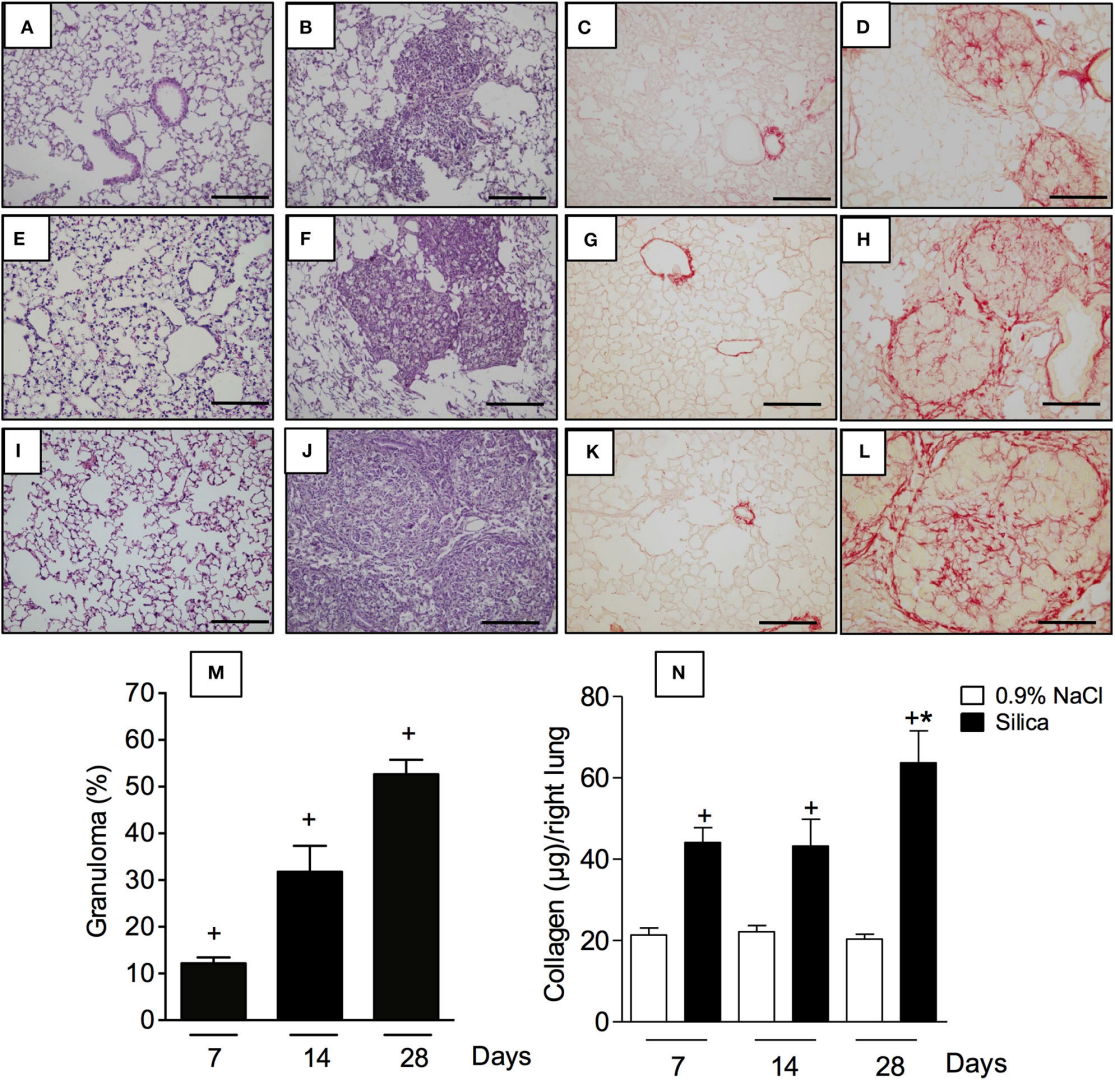
Pathological changes caused by silica particle instillation. Lung sections were obtained from sham-challenged mice **(A,E,I/C,G,K)** or silica-challenged mice **(B,F,J/D,H,L)** (**H,E**/Picrus Sirius staining) on days 7, 14, and 28, respectively. Quantitative evaluation of area occupied by granuloma and lung collagen content are seen in **(M)** and **(N)**, respectively. Scale bar = 200 μm. Values represent mean ± SEM from 5 animals per group. Statistical analysis was done with one-way ANOVA followed by Newman-Keuls-Student test. ^+^*P* < 0.05 as compared to saline-challenged animals. *P < 0.05 as compared to silica-challenged animals.

In order to evaluate the impact of silica particle instillation in the lungs, tissue sections were examined under light microscopy. Specimens obtained from sham-challenged mice at days 7, 14, and 28, stained with either H&E (Figures 3A,E,I, respectively) or Picrus sirius (Figures 3C,G,K, respectively), showed no pathological changes as expected. In contrast, H&E-staining revealed a clear thickening of alveolar walls as well as progressive increase in areas of parenchyma occupied by granuloma in silica-challenged mice at day 7 (Figure 3B), day 14 (Figure 3F) and day 28 (Figure 3J) post-challenge. Quantitative morphometric analyses confirmed the time-dependent formation of granulomas in the lungs of silica-challenged mice (Figure 3M). In parallel, Picrus sirius staining of lung sections from silicotic mice revealed a time-dependent increase in the amount of collagen deposition in the parenchyma at day 7 (Figure 3D), day 14 (Figure 3H), and day 28 (Figure 3L). These histological findings close-correlated with the levels of total collagen content in the lungs, quantified by the Sircol assay (Figure 3N). Silica challenge did not cause death of animals during the course of the experiments.

### Treatment With Flunisolide Inhibits Lung Inflammation, Fibrosis, and Airway Hyper-Reactivity in Silicotic Mice

**Figure 4:**
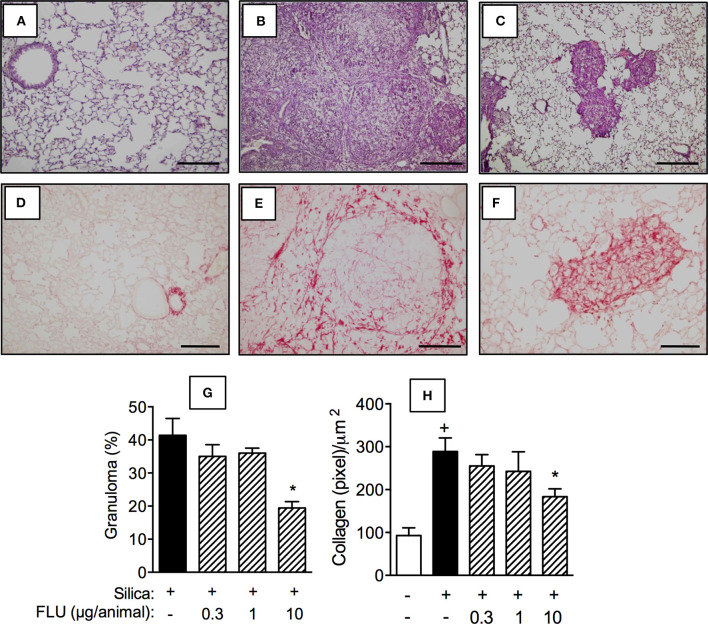
Effect of flunisolide on granulomatous and fibrotic response caused by silica particle instillation. Lung sections were obtained from sham-challenged mice (negative control) **(A/D)**, silica-challenged mice treated with vehicle (positive control) **(B/E)** and silica-challenged mice treated with flunisolide (10 μg/mouse, intranasal, daily from days 21–27) **(C/F)** (**H,E**/Picrus sirius staining) on day 28. Quantitative evaluation of the area occupied by granuloma and lung collagen content from silicotic mice treated or not with flunisolide are seen in **(G)** and **(H)**, respectively. Scale bar = 200 μm. Values represent mean ± SEM from 6 animals per group. Statistical analysis was done with one-way ANOVA followed by Newman-Keuls-Student test. ^+^*P* < 0.05 as compared to saline-challenged animals. *P < 0.05 as compared to silica-challenged animals.

Interventional local treatment with flunisolide (10 μg/mouse), given daily from day 21 to 27, via nasal instillation, reduced both granulomatous response (Figure 4C) and collagen deposition (Figure 4F) caused by silica particles as compared to untreated silicotic mice, concerning granuloma formation (Figure 4B) and collagen deposition (Figure 4E), respectively. Flunisolide treatment reduced, but did not abolish, these changes as can be attested by the comparison with lung sections from negative controls (Figures 4A,D). Quantitative values are shown in Figures 4G,H for granulomatous and fibrotic response, respectively. There was no effect on silica-induced granulomatous and fibrotic response when flunisolide was given at the doses of 0.3 or 1 μg/mouse.

**Figure 5:**
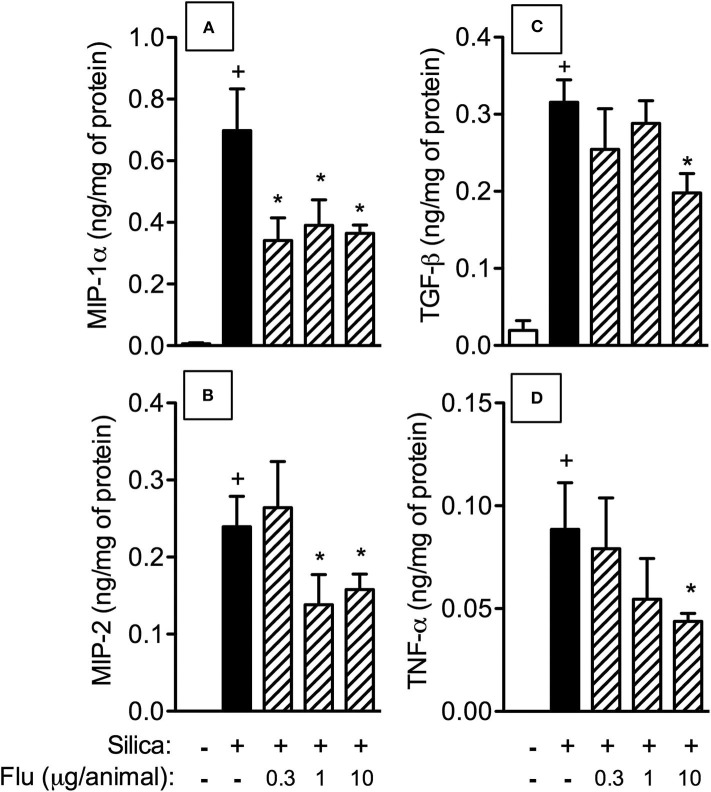
Effect of flunisolide on generation of inflammatory mediators caused by silica particle instillation. Chemokines MIP-1α/CCL3 **(A)**, MIP-2/CXCL2 **(B)** and cytokines TGF-β **(C)** and TNF-α **(D)** were measured in the lung tissue obtained from sham-challenged mice (negative control), silica-challenged mice treated with vehicle (positive control) and silica-challenged mice treated with flunisolide (0.3–10 μg/mouse, intranasal, daily from days 21–27) on day 28. Values represent mean ± SEM from 6 animals per group. Statistical analysis was done with one-way ANOVA followed by Newman-Keuls-Student test. ^+^*P* < 0.05 as compared to saline-challenged animals. *P < 0.05 as compared to silica-challenged animals.

We then examined the effect of flunisolide (0.3, 1 and 10 μg/mouse) on pro-inflammatory and pro-fibrotic mediators generated in the lung tissue of silicotic mice. Figure 4 shows that silica particle exposure upregulated the levels of MIP-1α/CCL-3 (Figure 5A) and MIP-2/CXCL-2 (Figure 5B), TNF-α (Figure 5C), and TGF-β (Figure 5D), all of which being partially inhibited by flunisolide (10 μg/mouse). As shown in this figure, doses as low as 0.3 μg/mouse and 1 μg/mouse inhibited MIP-1α/CCL-3 and MIP-2/CXCL-2, respectively, but did not affect the cytokines.

**Figure 6:**
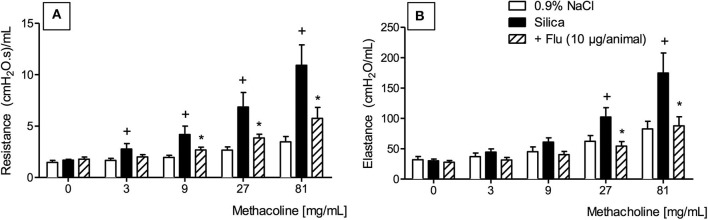
Effect of flunisolide on airway hyper-reactivity caused by silica particle instillation. Airway resistance **(A)** and pulmonary elastance **(B)** were evaluated in the presence of increasing concentrations of methacholine (3–81 mg/mL) in sham-challenged mice (negative control), silica-challenged mice treated with vehicle (positive control) and silica-challenged mice treated with flunisolide (10 μg/mouse, intranasal, daily from days 21–27) on day 28. Values represent mean ± SEM from 6 animals per group. Statistical analysis was done with one-way ANOVA followed by Newman-Keuls-Student test. ^+^*P* < 0.05 as compared to saline-challenged animals. *P < 0.05 as compared to silica-challenged animals.

We then employed invasive barometric plethysmography to assess whether flunisolide could repair the lung function disorder caused by silica particle exposure. As shown in Figure 6, silicotic mice reacted with exacerbation of the increase in airway resistance (Figure 6A) and lung elastance (Figure 6B) caused by aerosolized methacholine. Intranasal flunisolide, administered daily from days 21 to 27, significantly inhibited silica-induced airway hyper-reactivity concerning both airway resistance (Figure 6A) and lung elastance (Figure 6B).

### Treatment With Flunisolide Improves Clearance of Silica Particles From the Lungs

**Figure 7:**
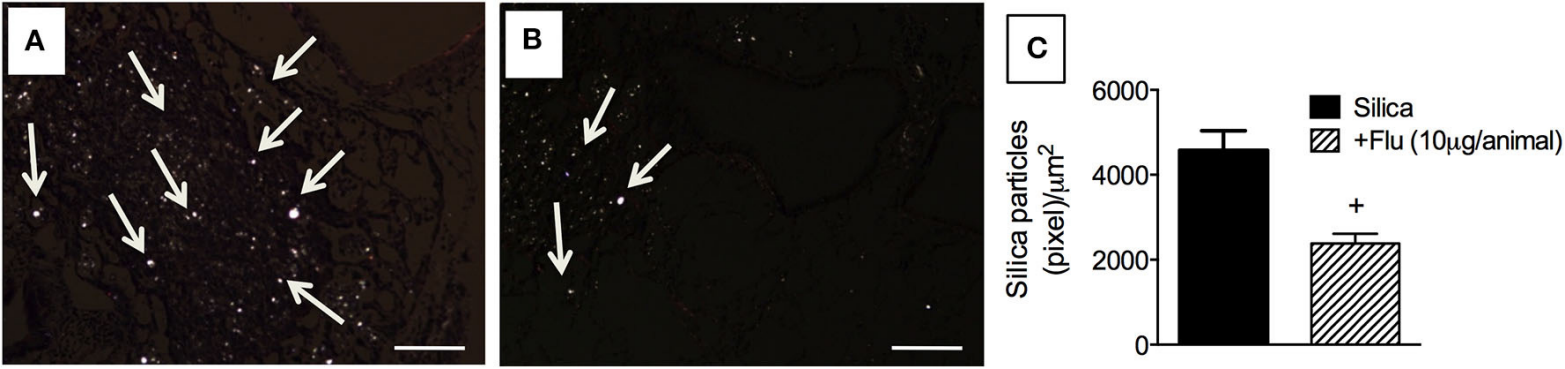
Effect of flunisolide on the presence of crystalline silica particles in the lungs. Tissue sections were obtained from silica-challenged mice treated with vehicle (positive control) **(A)** and silica-challenged mice treated with flunisolide (10 μg/mouse, intranasal, daily from days 21–27) **(B)**. Quantitative evaluation of silica particles is seen in **(C)** (Picrus sirius staining/polarized microscopy). Arrows indicate silica particles. Scale bar = 200 μm. Values represent mean ± SEM from 6 animals per group. Statistical analysis was done with one-way ANOVA followed by Newman-Keuls-Student test. ^+^*P* < 0.05 as compared to saline-challenged animals.

Residual silica particles present inside the lungs trigger and perpetuate inflammation and development of fibrosis. By means of polarized light, crystalline silica particles can be visualized based on their property to exhibit birefringence (19). By means of polarized microscopy, we detected the presence of small bright crystals in the lungs of silica-challenged mice (Figure 7A), and that treatment with flunisolide (10 μg/animal) reduced the number of these particles (Figure 7B). Quantitative data are shown in Figure 7C.

### Treatment With Flunisolide Inhibits Silica-Induced Macrophage and Myofibroblast Accumulation in the Lung Tissue

**Figure 8:**
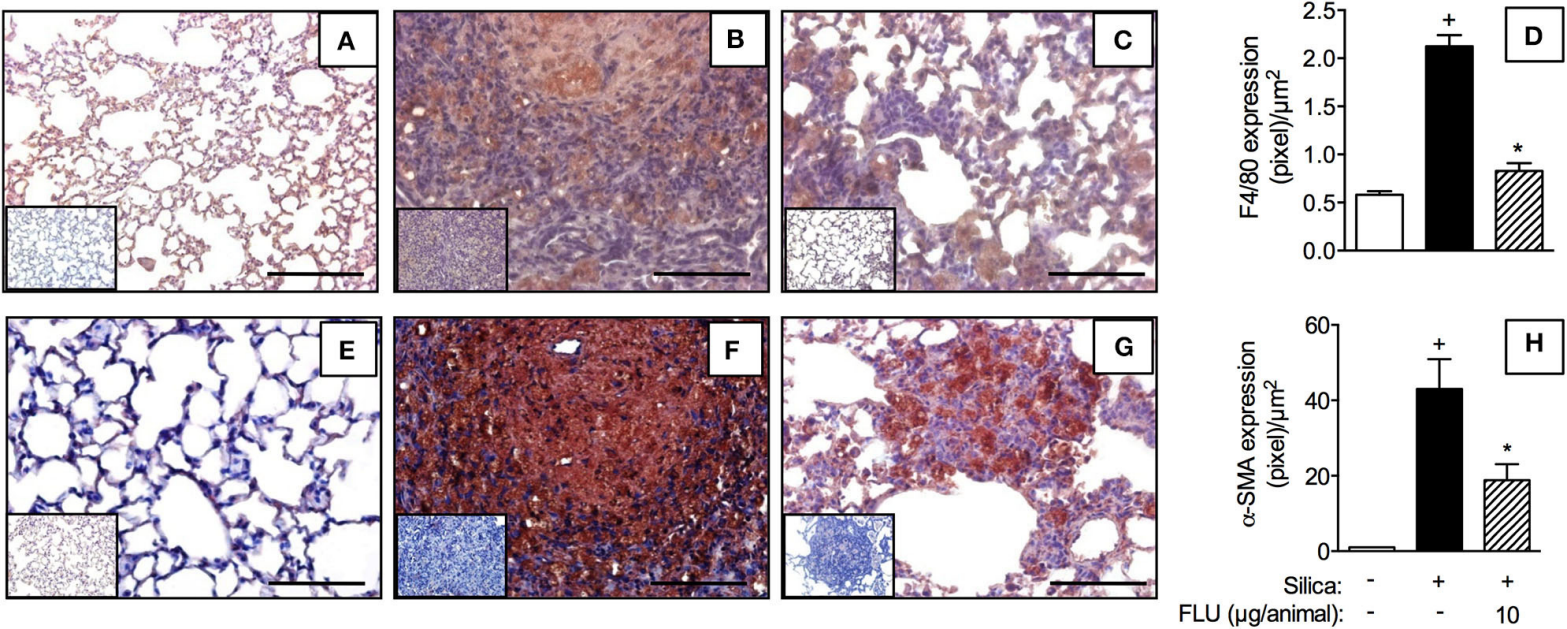
Effect of flunisolide on accumulation of macrophages and myofibroblasts caused by silica particle instillation. Lung sections were obtained from sham-challenged mice (negative control) **(A,E)**, silica-challenged mice treated with vehicle (positive control) **(B,F)** and silica-challenged mice treated with flunisolide (10 μg/mouse, intranasal, daily from days 21–27) **(C,G)** (F4/80 and α-SMA imunohistochemical labeling) on day 28. Measurements of pixels per micrometer square for F4/80 and α-SMA positive cells are shown in **(D)** and **(H)**, respectively. IHC negative control (inset) consisted in the absence of primary antibody. Scale bar = 200 μm. Values represent mean ± SEM from 6 animals per group. Statistical analysis was done with one-way ANOVA followed by Newman-Keuls-Student test. ^+^*P* < 0.05 as compared to saline-challenged animals. *P < 0.05 as compared to silica-challenged animals.

In comparison to sham-challenged mice (Figures 8A,E), the silicotic ones presented increased lung tissue levels of macrophages (Figure 8B) and myofibroblasts (Figure 8F), as revealed by F4/80 and α-SMA immunelabelling, respectively. Flunisolide (10 μg/mouse) given daily from day 21 to 27 reduced the number of F4/80 (Figure 8C) and α-SMA positive cells (Figure 8G). Quantitative data for F4/80 and α-SMA positive cells are shown in Figures 8D,H, respectively.

### Treatment With Flunisolide Inhibited Lung Fibroblast Activation *in vitro*

**Figure 9:**
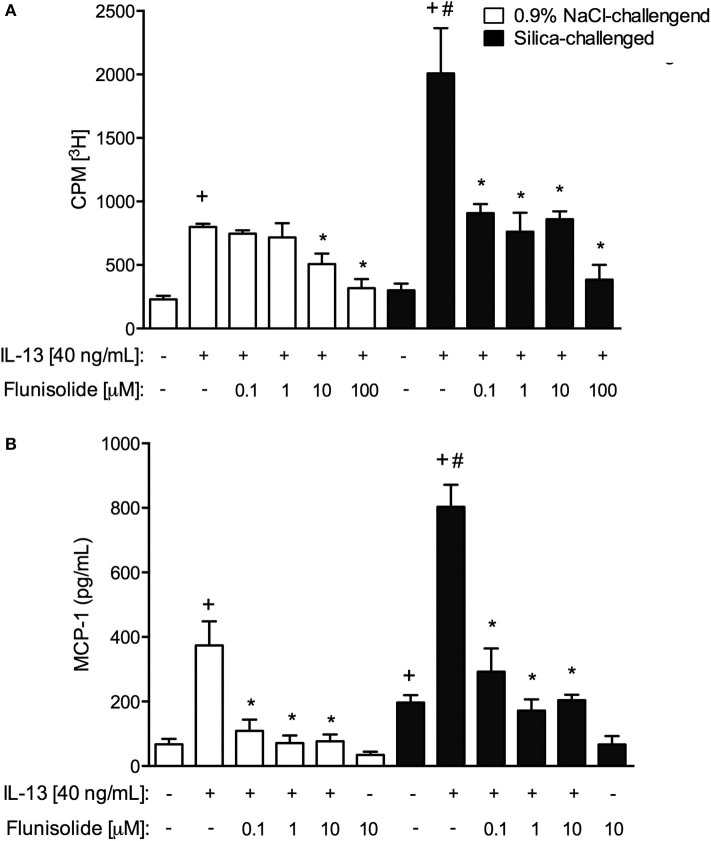
Proliferation **(A)** and chemokine MCP-1 production **(B)** in cultured lung fibroblasts recovered from normal (white columns) and silica-challenged mice (black columns). Cells were exposed with flunisolide (Flu) (0.1–100 μM), 1 h before stimulation with IL-13 (40 ng/mL), and the analysis was performed 24 h later. Values represent mean ± SEM of quadruplicates from 3 independent experiments. Statistical analysis was done with one-way ANOVA followed by Newman-Keuls-Student test. ^+^*P* < 0.05 compared to respective medium-stimulated group; ^#^*P* < 0.05 compared to IL-13-challenged normal group; *P < 0.05 compared to respective IL-13-challenged group.

Finally, we examined the capacity of flunisolide to directly modulate the IL-13-induced activation of fibroblasts *in vitro*. Cells were recovered from the lungs of saline- and silica-challenged mice, and proliferative (Figure 9A) and secretory (Figure 9B) activities were evaluated. Incubation of normal fibroblasts with the pro-fibrotic cytokine IL-13 (40 ng/mL) triggered proliferation and MCP-1/CCL2 production (Figures 9A,B, respectively). Interestingly, silicotic fibroblasts showed increased baseline levels of MCP-1/CCL-2 production as compared to those from normal controls (Figure 9B). Stimulation with IL-13 increased proliferation and MCP-1/CCL-2 production in silicotic fibroblasts at higher levels when compared to control fibroblasts, suggesting a primed phenotype. Fibroblasts from saline- and silica-challenged mice showed IL-13-induced proliferation and MCP-1/CCL-2 production, responses significantly reduced following incubation with flunisolide (0.1 to 10 μM) (Figure 9).

## Discussion

The current study addressed the effect of therapeutic treatment with flunisolide on silica-induced pulmonary inflammation and fibrogenesis in mice. The nasal instillation of crystalline silica promoted a pulmonary fibro-granulomatous response, starting as lung inflammatory process and evolving to form granuloma rich in collagen fibers. Flunisolide inhibited granuloma formation and collagen deposition induced by silica in the mouse lungs. This effect appeared related to the decrease in the content of F4/80 and α-SMA positive cells in the lungs, which paralleled with reduction of pro-inflammatory and pro-fibrotic cytokines and chemokines. Flunisolide also suppressed IL-13-induced proliferation and MCP-1/CCL-2 release from lung myofibroblasts recovered from saline- and silica-challenged mice. These findings indicate that flunisolide seems to be an encouraging therapeutic strategy to be used in the case of silicosis.

Our current understanding of the precise mechanistic connection between inflammation and fibrogenesis is limited, in spite of intensive research in the field (20). Existing evidence strongly suggests that in the lung, as well as in many other tissues, fibrosis is underlined by a complex multistep inflammatory response driven by a network of cytokines/chemokines, growth factors and signaling processes, often associated with leucocyte infiltration and fibroblast activation (20, 21). In the murine model of silicosis used in this study, Swiss Webster was the mouse strain of choice as this is not an inbred mouse strain, thereby modeling the outbred human condition associated with clinical silicosis as previously reported (15, 22–24). The kinetics of leukocyte infiltration in BAL fluid revealed that there was a marked increase in the number of total leukocytes at 7 days, which reduced progressively with time. A similar profile was noted in the case of mononuclear cells, while neutrophil numbers remained elevated for at least 28 days after nasal instillation of silica into mice. The latter paralleled with the increased levels of KC/CXCL-1 detected in the BAL of silica challenged-mice. Tissue analyses showed that there was a marked inflammatory cell infiltration at day 7, peribronchiolar granuloma formation at day 14, followed by an extensive tissue fibrosis 28 days post-silica challenge. In parallel, a marked increase in the levels of fibrogenic cytokines namely TGF-β and TNF-α was detected in both BAL and lung tissue. These responses directly correlated with alteration of lung function as attested by the state of airway hyper-reactivity to the spasmogenic methacholine.

Despite the sustained incidence of silicosis, little has been achieved concerning treatment development. Studies aiming to identify an effective therapy for silicosis are occurring, though management of silicosis still consists of supportive therapy including the use of bronchodilators and cough medication, as well as oxygen therapy (25–27). We demonstrated, herein, the efficacy of the therapeutic administration of flunisolide in reversing granuloma formation and collagen deposition triggered by silica particles. Our results are in line with those which showed glucocorticoids inhibiting remodeling and fibrosis in experimental models of lung allergic inflammation (13) and carbon tetrachloride-stimulated spleen in mice (28). Our findings are also supported by earlier clinical studies showing acute and chronic silicosis somehow responding to glucocorticoid therapy (11, 29, 30). Glucocorticoids are known for their ability to down-regulate inflammatory signaling, a response attributed to repression of the transcription of target pro-inflammatory genes through inhibition of nuclear factor-κB (NF-κB) and activator protein-1 (AP-1) activation (31). Consistently, treatment with flunisolide effectively inhibited cytokine generation as well as labeling of α-SMA-positive fibroblasts into granulomatous areas in lungs of silicotic mice, phenomena that can be correlated to the reduction of pro-fibrotic cytokine generation as TGF-β and TNF-α.

A lot of attention has been directed toward myofibroblasts as central cells in the development of lung fibrosis based on their capacity to generate extracellular matrix components (32), thus being considered important targets for antifibrotic drugs. We also assessed the direct effect of flunisolide on lung fibroblasts recovered from normal or silica-challenged mice in the presence and absence of IL-13. Interestingly, the basal levels of MCP-1/CCL-2 generated in the case of fibroblasts from silica-stimulated mice were significantly higher when compared to normal mice, suggesting a change in the cell phenotype. MCP-1/CCL-2 is a chemokine known to up-regulate collagen gene expression (33), being implicated in lung fibrosis. We detected increased levels of MCP-/CCL-2 in the lungs of silicotic mice already on day 7 post-challenge (24), suggesting that the chemokine might be implicated in the lung fibrotic response to silica crystals. It was reported that fibroblasts are key cells involved not only in physiological but also several pathological processes (34) including fibrosis, when they can be activated and change their phenotype, particularly after being in contact with pro-fibrotic cytokines including TGF-β (35) and IL13 (34). We found that flunisolide inhibited fibroblast proliferation as well as MCP-1/CCL-2 production *in vitro*, from both normal and silica-challenged mice. These data can help to explain the reduction of the granulomatous response and labeling of α-SMA-positive fibroblasts noted in the flunisolide-treated silicotic mice.

Macrophages also play an important role in regulating the fibrotic process, based on their phagocytic activity (36, 37). Inhalation of crystalline silica is a persistent process that reflects multiple ingestion and re-ingestion cycles of particles by macrophages, causing activation and release of various pro-inflammatory and pro-fibrotic factors, resulting in sustained inflammation and ultimately fibrosis (38). We found that treatment with flunisolide reduced the number of F4/80 positive cells and levels of lung cytokines (TNF-α and TGF-β) and chemokines (MIP-1α/CCL-3 and MIP-2/CXCL-2), suggesting that mediators released by macrophages were inhibited by flunisolide. Down-regulation of macrophage functionality can be associated with the effect of glucocorticoids on the cytoskeleton, leading to reduction of cell adherence and/or migration to the inflammatory site (39–43) or even induction of apoptosis (44–46), thus contributing to the inhibition of fibrosis by flunisolide. Alternatively, macrophages that have taken up silica particles could be carried off the lungs by the lymphatics (15). Although silicosis is primarily a lung-associated disease, it also affects draining intrathoracic lymph nodes (47). In parallel to typical silicotic alterations in the lungs, changes in the mediastinal lymph nodes were already described including fibrosis and the presence of silica particles. Our group reported that amelioration, by the immunotoxin IL-13PE, of the granulomatous fibrosis in silica-challenged mice, was clearly associated with the reduction in the amount of free silica particles present in the lung parenchyma (15). In the current study, treatment with flunisolide showed a marked tendency for reduction in the amount of silica particles in the lungs. Moreover, the reversal of lung granulomatous fibrosis caused by flunisolide could lead to an enlargement of alveolar spaces, supporting the idea that the treatment might be acting in favor of the exhalation of free silica particles through the airways.

Finally, previous reports stated that patients with silicosis exhibit a respiratory deficit and airway hyper-reactivity (48), and in more severe cases increased airways resistance and residual volume were reported (6, 49, 50). By means of whole-body invasive plethysmography, it became evident that flunisolide decreased silica-induced state of airway hyper-reactivity, as attested by the reduction in lung resistance and elastance following methacholine aerosolization. It is noteworthy that TNF-α is implicated in airway hyper-reactivity (51), adding support to the interpretation that the suppressive effect of flunisolide on airway hyper-reactivity and other pathological features of silicosis is at least in part accounted for by a decrease in the generation of cytokines.

Since silicosis is a chronic inflammatory disfunction, this study had its inherent limitations in being based in a short-term murine model of the disease, imposed in some extent by ethical restrictions concerning the use of laboratory animals. In addition, although the findings from this study pointed out the positive influence of the flunisolide interventional treatment, the lack of data on the persistence of the protective effects on time-points later than 28 days post-challenge is also a limitation.

Overall, our findings show that therapeutic treatment with the intranasal glucocorticoid flunisolide improved granulomatous inflammation and fibrosis, leading to restoration of lung function in silica-challenged mice. They also indicate that local flunisolide seems to constitute a promising therapeutic alternative for treatment of lung fibrotic diseases such as silicosis.

## Data Availability Statement

All datasets generated for this study are included in the article/supplementary material.

## Ethics Statement

The animal study was reviewed and approved by Committee on Use of Laboratory Animals of the Oswaldo Cruz Foundation (license LW057/14).

## Author Contributions

TF, JL, AA, FF-F, FG, and YJ: data acquisition, analysis and interpretation of data for the work, provided illustration. VC: drafting of the work. JW and CH: revising it critically for important intellectual content. MM: substantial contributions to design of the work, revising it critically for important intellectual content, financing. PS: design of the work, supervision and final approval, financing, agreement to be accountable for all aspects of the work in ensuring that questions related to the accuracy or integrity of any part of the work are appropriately investigated and resolved. All authors contributed to the article and approved the submitted version.

## Conflict of Interest

The authors declare that the research was conducted in the absence of any commercial or financial relationships that could be construed as a potential conflict of interest.

## Abbreviations

AHR: airway hyper-reactivity
α-SMA: α-smooth muscle actin
BAL: bronchoalveolar lavage
NF-κB: nuclear factor-κB
AP-1: activator protein-1
MCP-1: monocyte chemoatractive protein-1
MIP-1α: monocyte inducible protein-1 alpha
MIP-2: monocyte inducible protein-2
TNF-α: tumor necrosis factor-alpha
TGF-β: tumor growth factor-beta
F4/80: also known EGF-like module-containing mucin-like hormone receptor-like 1 (EMR1)
HRP: horseradish peroxidase
Tris-HCl: tris (hydroxymethyl) aminomethane hydrochloride
DMEM: Dulbecco's Modified Eagle Medium
IL-13: interleukin-13
IL-13PE: Interleukin-13 conjugated to mutated *Pseudomonas aeruginosa* exotoxin.
